# The "hypnotic state" and eye movements: Less there than meets the eye?

**DOI:** 10.1371/journal.pone.0182546

**Published:** 2017-08-28

**Authors:** Etzel Cardeña, Barbara Nordhjem, David Marcusson-Clavertz, Kenneth Holmqvist

**Affiliations:** 1 Center for Research on Consciousness and Anomalous Psychology (CERCAP), Department of Psychology, Lund University, Lund, Sweden; 2 Laboratory for Experimental Ophthalmology, School of Behavioural and Cognitive Neurosciences, University Medical Center Groningen, Groningen, The Netherlands; 3 Pennsylvania State University, Department of Biobehavioral Health, University Park, PA, United States of America; 4 Eye-tracking Group, Humanities Laboratory, Lund University, Lund, Sweden; 5 UPSET, NWU Vaal, Vanderbijlpark, South Africa; University of Melbourne, AUSTRALIA

## Abstract

Responsiveness to hypnotic procedures has been related to unusual eye behaviors for centuries. Kallio and collaborators claimed recently that they had found a reliable index for "the hypnotic state" through eye-tracking methods. Whether or not hypnotic responding involves a special state of consciousness has been part of a contentious debate in the field, so the potential validity of their claim would constitute a landmark. However, their conclusion was based on 1 highly hypnotizable individual compared with 14 controls who were not measured on hypnotizability. We sought to replicate their results with a sample screened for High (n = 16) or Low (n = 13) hypnotizability. We used a factorial 2 (high vs. low hypnotizability) x 2 (hypnosis vs. resting conditions) counterbalanced order design with these eye-tracking tasks: Fixation, Saccade, Optokinetic nystagmus (OKN), Smooth pursuit, and Antisaccade (the first three tasks has been used in Kallio et al.'s experiment). Highs reported being more deeply in hypnosis than Lows but only in the hypnotic condition, as expected. There were no significant main or interaction effects for the Fixation, OKN, or Smooth pursuit tasks. For the Saccade task both Highs and Lows had smaller saccades during hypnosis, and in the Antisaccade task both groups had slower Antisaccades during hypnosis. Although a couple of results suggest that a hypnotic condition may produce reduced eye motility, the lack of significant interactions (e.g., showing only Highs expressing a particular eye behavior during hypnosis) does not support the claim that eye behaviors (at least as measured with the techniques used) are an indicator of a "hypnotic state.” Our results do not preclude the possibility that in a more spontaneous or different setting the experience of being hypnotized might relate to specific eye behaviors.

## Introduction

Responsiveness to hypnotic procedures has been related to unusual eye behaviors for centuries. Kallio et al. [1] claimed that they had found a reliable index for "the hypnotic state" in eye behaviors evaluated through eye-tracking techniques (see also https://www.newscientist.com/blogs/nstv/2011/10/one-word-technique-produces-first-physical-evidence-of-hypnosis.html). Whether hypnotic responding involves a special state of consciousness (or special psychological processes), with concomitant neurophysiological changes, or whether it can be explained fully by more common psychological processes has been debated for centuries [2]. Many theoreticians in the field do not take extreme positions in this issue and integrate both approaches, but finding a sensitive and specific neurophysiological indicator of general hypnotic responding would be an important contribution to the field [3]. However, Kallio et al.'s conclusion can be questioned on various substantive and methodological grounds. In this paper, we review the literature on hypnosis and eye movements, focusing on the research by Kallio et al., and report a study with a larger *N* and other methodological changes that could more clearly evaluate their claim.

## Hypnosis and eye movements

Throughout much of its history, alterations of consciousness and behavior related to hypnosis and related phenomena have been associated with unusual ocular behavior. For instance, Charcot and Richer [4] collected depictions of “spirit possessed” individuals, many of whom showed either goggle- or rolled-up eyes, whereas Sargent [5–6] presented photographs of similar expressions among members of snake handling and spirit possession groups. There was a short step from spirit possession and exorcism to mesmerism, the precursor of hypnotic techniques. In 1775, Franz Anton Mesmer showed that he could elicit and terminate spirit possession signs through the use of hand touch and passes, which he explained in secular terms as involving animal magnetism rather than devils [7].

Even after the main explanation for hypnotic phenomena became the psychological effect of suggestions rather than an undetectable *animal magnetism*, unusual ocular behavior continued to be mentioned as an indicator of a putative hypnotic state. The first major historical figure to develop this connection was the Scottish physician James Braid in the mid-19th century. He emphasized eye fixation and the inability to open the eyes as markers of a hypnotic state and favored an induction involving a strained upwards fixation of the eye and suppressed breathing [8]. Also involving a strained upwards movements of the eyes, H. Spiegel and D. Spiegel [9] stated that an indicator of an "inherent potential capacity for experiencing hypnosis" (p. 54) is the ability to roll the eyes upwards (or ERS for eye-roll sign), particularly if they cross as they go up. However, the ERS has only weak positive correlations with a related measure of hypnotizability, [10]. Nonetheless, movies and other popular depictions have typically depicted individuals "in trance” with an unblinking, unfocused gaze.

### Eye movement recordings during hypnosis

Starting in the 1960s, André Weitzenhoffer conducted a number of studies on some aspects of eye behavior and hypnosis. He reported that during hypnosis spontaneous eye-blink rate (BR) decreased significantly and strongly (to less than half) in people scoring in the upper half of hypnotizability (i. e., 6 or above in a scale of 0–12) but not in those in the lower half [11]. The method to measure BR was not specified, however, other than mentioning “a simple ophthalmological examination” (p. 672), which we interpret as manual counting rather than automatic recording.

In a probable extension of that study, Weitzenhoffer [12] tested 43 participants in a design including different types of induction and a group of hypnotic simulators, with specific ophthalmological tests: manual recording of BR and motility, an optokinetic nystagmus (OKN), and a convergence pursuit test. He reported that some hypnotized individuals hardly moved their eyes and that the more usual response was decreased BR and eye motility, particularly among those more highly hypnotizable (p. 109), and that these effects were accentuated when he used an eye fixation induction. He concluded that "reductions in blink rate, motility, and OKN… appear to be reasonably… intrinsic to hypnosis and… the 'trance stare'” (p. 110).

In another study, Weitzenhoffer [13] placed electrodes on the outer canthus of the eyes in a sample of 15 participants, but it should be noted that electro-oculography (EOG) produces a noisy signal and has poor accuracy and problems correcting for eye drifts baseline. He found that large, saccadic eye movements occurred among participants with eyes open or closed outside of a hypnotic context (which he called REM but were probably just ordinary saccades). However, induction of hypnosis with an eye fixation technique seemed (no quantitative analyses were reported) to produce very small eye movements (which he termed micro eye movements or MEM, not to be confused with microsaccades); the higher hypnotizables also produced spontaneously what he called slow eye movements (SEM, not to be confused with smooth eye pursuit). Tebecis and Provins [14] replicated a decrease in REM and increase of SEM, in addition to upward eye-rolling and “eye fluttering” during hypnosis following specific suggestions to imagine a train trip or a tennis match, but mentioned considerable intra-group variability. Other investigators [15] did not replicate an increase in SEM during hypnosis when comparing it to relaxation, nor did they find a relation between SEM and level of hypnotizability, but provided very little descriptive or inferential statistics information so their results cannot be clearly evaluated. There have also been more recent studies with advanced eye-tracking technology. A study replicated a decrease in BR for high hypnotizables (henceforth Highs) but not for low hypnotizables (henceforth Lows) during an auditory vigilance task under hypnosis [16]. Lichtenberg et al. [17] tested a proposed link between central dopaminergic activity and hypnotizability by evaluating BR across different levels of hypnotizability (BR has been positively related to striatal dopamine activity [18]). Contrary to their expectation, they found a higher BR among Medium hypnotizables than among Highs, but it should be mentioned that none of the conditions they tested included a hypnotic procedure, which might have shown a different effect for BR across different hypnotizability levels.

**Fig 1:**
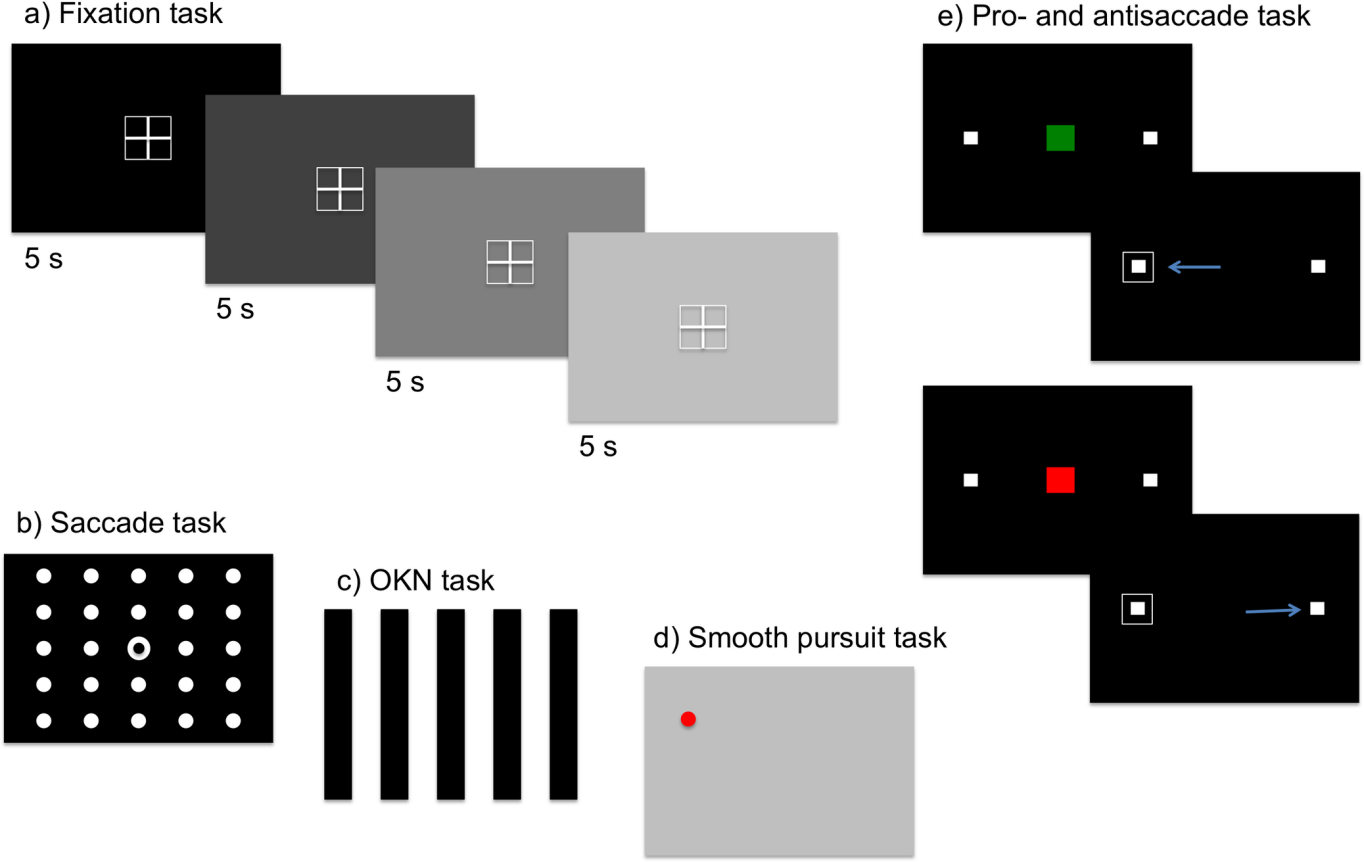
Depiction of the experimental tasks. Fixation task (a), participants maintained fixation at a central location while the luminosity of the background changed. Saccade task (b), each trial began with a central fixation point followed by a target that could appear at one of the locations in a 5x5 matrix covering the screen. OKN task (c), participants were asked to look at the centre of the screen while viewing a drifting grating. Smooth pursuit task (d), participants followed a moving dot with their eyes. Pro- and antisaccade task (e), a central marker indicated a pro (green) or an anti (red) trial. In prosaccade trials, the task was to make a saccade to the framed square, while in antisaccade trials it was to look towards the unframed square. The arrows illustrate the correct target in pro- and anti-saccade trials.

In a study [19] that used a rest (not hypnosis) condition, Highs started with a higher BR than medium and low hypnotizables (these two groups did not differ from each other) during the first minute of the session, but their BR progressively decreased during the following minutes. Although BR decreased for all levels of hypnotizability, inspection of the study's Fig 1 suggests that there might have been a significant interaction (the authors did not report such analysis) in that the decrease of BR appears to have been greater among Highs than among the other groups. When the authors adjusted for mind wandering there were no longer hypnotizability differences in BR, suggesting that BR correlates with mind wandering, as was found in another study [20]. Di Gruttola et al. [19] wrote that at that time no difference had been found in mind wandering among hypnotizable groups, but a later study found more spontaneous mind wandering/daydreaming in Highs than in Lows [21]. Thus, the higher rate of BR among Highs may be mediated by how much they mind wander. With respect to dissociation, which has been measured in some studies along with hypnotizability, the subgroup of those who are highly dissociative report more detachment and lower cognitive control than those who are not [21].

Finally, in the study mentioned earlier [1], a High was compared with a control group that tried to imitate her behavior in the three eye-tracking tasks they considered optimal for their study: fixation, saccade, and OKN. Kallio et al. concluded that during the hypnotic condition, their single High (TS-H) differed from the control group in: exhibiting reduced eye blinking during the fixation task, although "some control subjects could mimic rather well this external feature" (p. 3); having a smaller pupil size and performing only short saccades towards the target in the saccade task; and in the OKN task having a decrease in number, size, and speed of ocular movements, and an increase in eye fixation. With respect to the OKN reported result, the smaller pupil size suggests a state of lower arousal. The sympathetic system, which dilates the pupil, generally activates fight-or-flight responses (increased heart rate, breathing, etc.), whereas the parasympathetic system, which constricts the pupil, generally conserves and restores resources in the body. Furthermore, there is a tight link between activation of the locus coeruleus and pupil diameter; and the locus coeruleus regulates the stress hormone norepinephrine, a central component in the fight-or-flight response [18, 22–23].

There are, however, a number of conceptual and design issues with Kallio et al.'s paper [1] that make the article's conclusion that it had "revealed” ocular indexes of a "hypnotic state” suspect. With respect to conceptual matters: 1) As reviewed above, the proposal of ocular markers of hypnosis is not new [12], although Weitzenhoffer was cautious about not reifying a "hypnotic state.” Nonetheless, Kallio et al. revisited some of Weitzenhoffer's proposals using more advanced technology. 2) There are a number of other indicators, both experiential and neurological, that have been previously found to differentiate between hypnotizable individuals in and out of hypnosis [24–25]), and could thus be seen as indexing a “state.” However, the concept of a “hypnotic state” (or any state of consciousness, for that matter) is limited insofar as it simplifies complex, dynamic processes that vary inter-individually and even intra-individually across different segments of the state [24, 26].

Besides the conceptual complexities of proposing a "hypnotic state," there are problematic design issues in the Kallio et al. study. First, they have published at least four other papers on TS-H, with three of them referring to, correctly in our view, a "case study.” We do not doubt that she qualifies as a hypnotic virtuoso, but we cannot know how representative she is of all other/many/some/a few/any other Highs. In fact, in a later study her performance following hypnotic suggestions differed markedly from that of another High [27]. In addition to her high hypnotizability, she might also vary in other psychological and neurological factors. For instance, the description by the authors that she immediately goes into a hypnotic state when the cue word "hypno” is mentioned is not typical of Highs, who experience themselves as being more hypnotized after an actual induction, typically much longer than a single word [28].

Second, the hypnotizability of the control group was not evaluated so it might have included a number of Highs, in which case the differences found would be irrelevant to hypnosis. It is customary in hypnosis research to compare task performance by level of hypnotizability. Third, the control group during the hypnosis condition was actually not in the same condition as TS-H. Whereas she carried out the eye-tracking tasks during a “waking” (preceded by the word "OK") and a hypnosis condition (preceded by the word "hypno”), the control group in the hypnosis condition was asked to simulate what TS-H had done during her "hypno” condition after watching films of her. Thus, this was not a comparison between a hypnotic condition of a High and that of non-Highs, but of the "hypno” condition of the High against the effortful simulation of a number of people with unevaluated hypnotizability.

Considering these issues, we decided to evaluate the Kallio et al. proposal with a within- and between-groups (mixed) design including Highs and Lows during hypnosis and waking conditions. We also added two tasks, which as far as we know have never been tested in a hypnosis study (all tasks are described in the Methods section). The first is an antisaccade task, a measure of inhibition of reflexive saccades, which has been used as a gross measure of prefrontal function [29] and attentional biases [30]. According to the theory that Highs in hypnosis exhibit flexible attention skills [31] we would expect Highs to perform better than Lows in this task. On the other hand, Woody and Bowers [32] would predict exactly the opposite because they have proposed that hypnotic performance subsumes a decrease of prefrontal functions. In that case we would expect to see a decrease in correct responses in the antisaccade task due to lower ability to inhibit reflex saccades [33–34] only or more markedly among Highs. The second added task, smooth pursuit, is affected by various psychological and neurological conditions, including schizophrenia or a predisposition to it, PTSD, and exposure to trauma [35–36] The addition of these two tasks was exploratory.

Our hypotheses follow, 2–4 are primarily based on Kallio et al.'s [1], whereas the remaining ones are based on other studies reviewed:

1. As a confirmation of the experimental manipulation of using a hypnosis induction, Highs will experience themselves as being more deeply hypnotized than Lows.
2. For the fixation task, during hypnosis BR will decrease for Highs (who will also exhibit a smaller pupil size), but show a smaller or no change for Lows. For the rest condition, there will be no differences.
3. For the saccade task, during the hypnosis -but not the rest- condition the Highs will exhibit fewer and shorter saccades than the Lows.
4. In the optokinetic nystagmus task, during hypnosis Highs will exhibit fewer and smaller saccades than the Lows. Because saccades and fixation duration are obviously strongly correlated (*r* = -.89 in our data), only the former will be reported. The groups were not expected to differ during the rest condition.
5. For the smooth pursuit task, Highs will show more catch-up saccades than Lows because they might daydream more.
6. Those reporting higher dissociative tendencies will also show more catch-up saccades because they are more likely to experience less cognitive control and more experiential detachment.

We did not have a specific hypothesis about the antisaccade task because the two theoretical hypnosis positions described above lead to opposite predictions, but the task was a good way to evaluate them.

## Materials and methods

### Design and participants

We used a 2 (high hypnotizability vs. low hypnotizability) x 2 (hypnosis vs. control sessions) counterbalanced order design. Level of dissociation was also evaluated. The mean age of the Highs (*n* = 16, 12 females) was 27.1 (*SD* = 10.62), whereas that of the Lows (*n* = 13, 5 females) was 26.8 (*SD* = 4.16) with 43% females. All participants were between 20–40 years old except for one 63-year old High, and they came from an ongoing database of more than 600 individuals who had been tested at that point for hypnotizability. Those who scored as Highs and Lows and were available participated in the study. There was no significant difference for age but the High group included significantly more females (*χ*^2^ = 3.94, *p* < .05).

### Instruments

Eye-tracking Tasks (Fig 1): The eye-tracking equipment was an SR EyeLink 1000 (SR Research, Ontario, Canada) in tower-mounted mode. Eye-tracking data were recorded at a sampling rate of 1000 Hz. Calibration and validation of eye fixation was carried out at the beginning of the hypnosis and control conditions using a nine-point procedure as implemented in the EyeLink software. Calibration was repeated when maximum error at validation was more than 1 degree. During all trials, participants had their head supported by a chin and forehead rest. We used an adjustable table set to the most comfortable level for each individual. Visual stimuli were presented on a Samsung SynchMaster LCD monitor with a refresh rate of 75 Hz (visual area measuring 375 x 300 mm, corresponding to 24.2 x 29.6 degrees). The resolution of the monitor was 1,280 pixels by 1,024 pixels. The experimental procedure for the *OKN*, *antisaccade* and *smooth pursuit* tasks were implemented in Matlab (R2011b), using the Psychophysics (PTB-3) and EyeLink Toolbox extensions [37–38]. The *fixation* and *saccade tasks* were implemented through the Experiment Builder (SR Research, Ontario, Canada).

In the *fixation task*, participants fixated their eyes on a central white target (a fixation cross presented inside a square frame of 2 x 2 degrees) shown on four different backgrounds that varied in luminosity (0, 30, 60, or 90% luminosity; 10 trials of each, presented in a random order, with a duration of 5–5.5 s each). Blink rates (blinks/s) and pupil sizes were measured for every background luminosity and averaged. The task lasted for ~ 4.5 min. The experiment parameters were reproduced from Kallio et al. [1].

In the *saccade task*, participants were instructed to gaze at white circular targets appearing on a black background as accurately and rapidly as possible. Trials started with a central white fixation point (shown for 100–500 ms) followed by the target circle, which could appear at 5 different eccentricities relative to the fixation point. The targets would each appear in a random location within a 5x5 matrix covering the screen (presented at ~ 4, 6, 8, 9, 12 degrees, targets at an eccentricity of 9 degrees appeared approximately twice as often as other eccentricities). There were a total of 75 trials presented in random order, and the task took less than 1 min. The experiment parameters were reproduced from Kallio et al. [1].

In the *optokinetic nystagmus task*, participants were asked to fixate on the center of the screen while a black and white grating moved towards the left or right side of the screen. Gratings moved for 8.5 s., either 4 or 6 degrees per sec. A total of 24 trials were presented in random order and the stimulus duration was ~ 3.4 min. The experiment parameters were reproduced from Kallio et al. [1] although we used 24 rather than 32 trials.

The *smooth pursuit task* involves asking the participant to visually follow a red circle moving across the screen horizontally, vertically, or in an ellipsis with a frequency between 0.2–0.5 Hz, extending over 20 degrees. The task consisted of 6 trials for a full cycle across the screen and it took ~1.2 min.

In the *antisaccade task*, participants were instructed to fixate on a central point until a target appeared. They had to then make a saccade towards (prosaccade) or away (antisaccade) from the target as quickly as possible. Targets could appear at an eccentricity of 5 or 10 degrees from the fixation point on the horizontal axis. The fixation point was presented for a random time between 800–1,200 ms followed by a 200 ms offset before another target was presented for 1,250 ms. A central fixation point indicated that it was a prosaccade when it changed to green, and an antisaccade when it turned to red. After the fixation point disappeared, two white squares appeared laterally on each side of the screen, equidistant from the center. There was a white frame around the target. Participants were given a practice run of 5 trials followed by an experimental run of 32 trials. The antisaccade task is used as a test of attention and top-down control, with failures in attention and control being associated with more errors [33–34]. The task lasted for ~2 minutes. We evaluated the percentage of correct responses and the saccade latency.

*Hypnotizability* was measured with the *Harvard Group Scale of Hypnotic Susceptibility* (HGSHS [39]) a widely used group measure of hypnotizability with good psychometric properties [40] and a strong correlation (*r* = .59) with the most demanding individual hypnosis scale [41]. It includes an induction followed by 12 suggestions referring to ideomotor, auditory hallucination, and post-hypnotic suggestion items; participants self-score their behavioral responses as pass or no pass. Those passing between 0–3 suggestions are considered Lows, those scoring between 9–12 items are considered Highs. In addition, 20 of the 29 participants had also filled out the *Subjective Experiences Scale* (SES [42]), a 12-item scale on a five-point format, with higher scores indicating a greater sense of involuntariness and experiential involvement in relation to the HGSHS suggestions. The SES seems to be a more sensitive indicator of the propensity to have alterations of consciousness than the HGSHS [43].

*Dissociation* was evaluated through the *Dissociative Symptoms Scale* (DSS), a valid and reliable measure of dissociative phenomena [44]. It consists of 20 items with five anchor points ranging from 0 (*not at all*) to 4 (*more than once a day*). Cronbach's alpha for this sample was .81.

*Hypnotic depth* was self-reported by participants using a scale from 0–10, in which "0" indicated being as awake and alert as possible, and "10" being as hypnotized as the person could get. Participants were asked to provide two ratings: the first after the hypnotic induction or the rest period (but before starting the eye-tracking tests) and the second for their “deepest” level of hypnosis after each condition. For the analyses, the ratings were summed so that the scores for each session could vary between 0 and 20. Self-ratings of hypnotic depth have been shown to be a valid and reliable indicator of hypnotic responses and spontaneous experiences [24, 45].

### Procedure

After the initial welcome to the experiment all the tasks were explained and shown on a monitor to familiarize the participants with them before exposure to the hypnosis or rest conditions. Each task was briefly explained again immediately before testing. The eye-tracker was calibrated for each participant by the second author. Then followed one of two conditions. The hypnotic condition (available from the first author) included an induction lasting about 4 min with eyes closed, with suggestions to relax the mind and the body and focus on the hypnotist's voice. Then followed progressive relaxation instructions to slightly tense various groups of muscles and then relax them while breathing out, along with a 1–20 count to increase hypnotic depth. These sets of suggestions were modeled after those used in the HGSHS and other standardized inductions used in hypnosis research but avoiding eye fixation suggestions. In the control condition, participants were requested to just relax with eyes closed for a minute or so but without using hypnosis or meditation. The first author served as hypnotist. In between eye-tracking tasks participants were told to stop supporting their chin on the stand if they so desired. In the hypnosis condition they were told to close the eyes and use the time to continue going deeply into hypnosis, whereas in the control condition they were told to close the eyes and just relax. In the hypnosis condition at the end of testing there was a 1–10 count with suggestions to come out of hypnosis. At the end of both conditions participants were asked if they felt alert and awake, and they all did.

The first two authors, who interacted with the participants, were masked to the hypnotizability level of the participants (the first author had conducted most hypnotizability testing in the past, but in groups of dozens and could not recall the hypnotizability of the participants other than in one or two cases). The research had obtained previous approval from the Regional Institutional Review Board and complied with its ethical guidelines. All participants were first explained the experimental procedure and allowed to ask questions, and signed a consent form.

### Analyses

Fixations and saccades were parsed online using the algorithm provided by SR Research. The saccade velocity was set to a threshold of 35 degrees/s and acceleration to 9,500 degrees/s^2^. For the analyses we only considered saccades larger than 1.5 degrees in the saccade, OKN, and pro- and anti-saccade tasks. Saccades with latencies below 80 ms were excluded from statistical analysis of the antisaccade task. For the smooth pursuit task, saccades larger than 10 degrees or with a duration over 100 ms were excluded. The pupil size is based on arbitrary units (a. u.) based on a relative measure by the eye tracker.

We conducted repeated measures ANOVAs to test for main effects and their interaction, and *t* tests for comparisons between groups. Because the groups differed in gender composition, we tested ANOVA models both with and without sex entered as a covariate. Because the results were virtually identical, we only report the results excluding sex for sake of brevity unless otherwise noted. We evaluated homogeneity of variance with Levene's test, and of covariance with Box's M test. Correlations were computed with *r* product moment correlations. The data did not violate normality assumptions so neither non-parametric analyses nor transformations were used. Partial eta squared and *d* were used as the effect size measures. The level of significance was set at *p* < .05 before conducting the analyses. Due to a technical error, there were missing data for four of the tasks. The statistical tests were carried out with the following number of participants: fixation task, *n* = 24; smooth pursuit task, *n* = 26; optokinetic nystagmus task, *n* = 26; saccade task, *n* = 23; pro- and antisaccade task, *n* = 29.

## Results

### Manipulation checks and order effects

We confirmed that our screening successfully yielded Highs and Lows with large differences in behavioral responses to the hypnotic suggestions and concomitant subjective experiences. Highs (*M* = 9.81, *SD* = 0.91) had much higher scores in the HGSHS than the Lows (*M* = 1.92, *SD* = 1.11), *t*(27) = 20.99, *p* < .001, *d* = 7.77. For those 20 who also completed the SES, the Highs (*M* = 44.86, *SD* = 6.07) had also reported greater involuntariness and automaticity during hypnosis testing than the Lows (*M* = 27.17, *SD* = 8.70), *t*(18) = 5.25, *p* < .001, *d* = 2.35. The Highs and Lows did not differ in dissociation (*t* (27) = .37).

**Fig 2:**
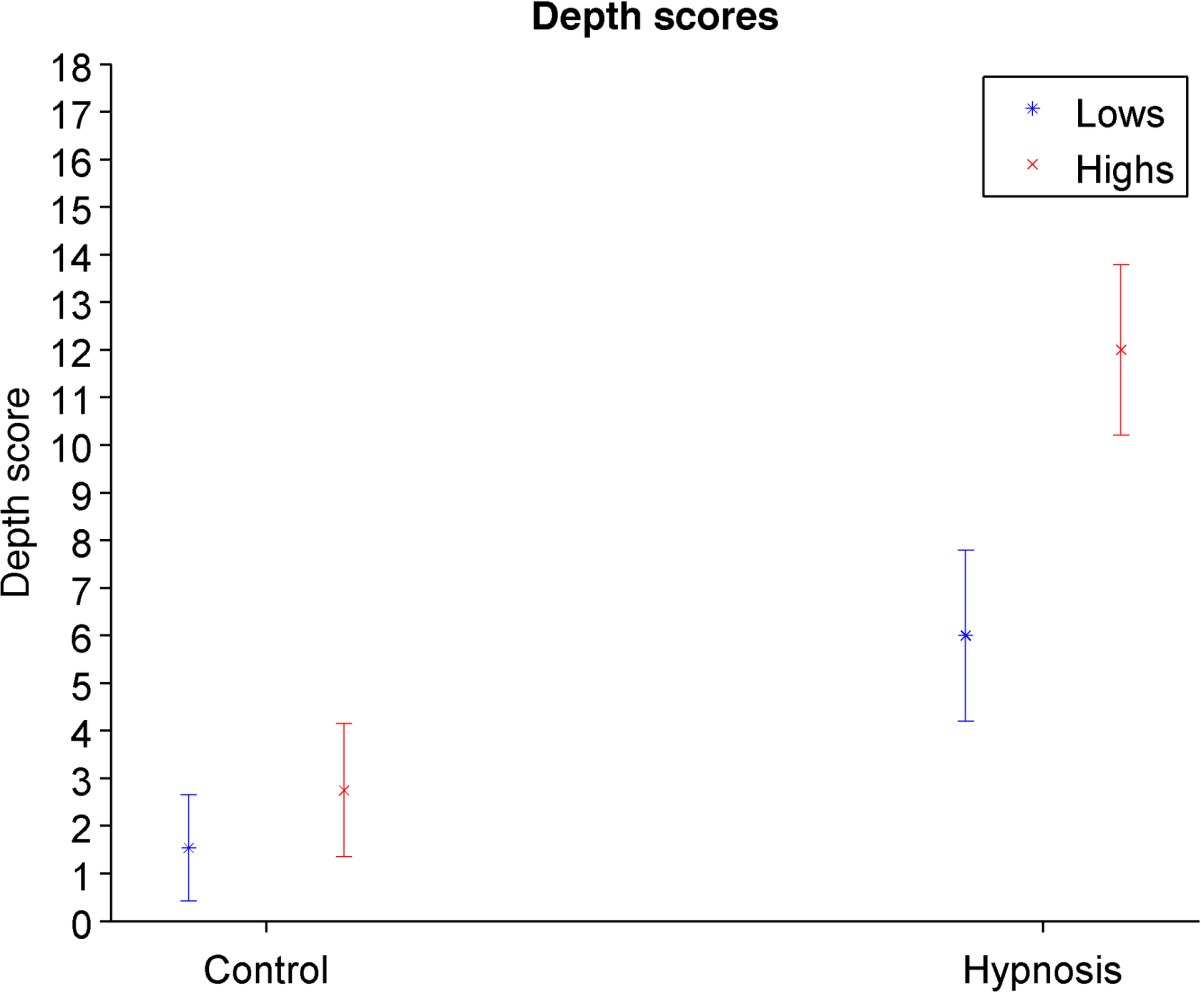
Reports of hypnotic depth per condition and hypnosis levels.

We next evaluated whether Highs experienced greater hypnotic depth than Lows during the sessions. Overall, participants reported greater hypnotic depth during the hypnotic than the control condition, *F*(1, 27) = 108.93, *p* < .001, *η*_*p*_^*2*^ = .80, and this effect was qualified by a two-way interaction between Condition and Hypnotizability, *F*(1, 27) = 13.28, *p* = .001, *η*_*p*_^*2*^ = .33. Highs (*M* = 2.75, *SD* = 2.62) and Lows (*M* = 1.54, *SD* = 1.85) did not differ significantly during the control condition, *t*(27) = 1.40, *p* = .17, *d* = 0.53, but Highs reported twice (*M* = 12.00, *SD* = 3.37) the hypnotic depth than Lows (*M* = 6.00, *SD* = 2.97) in hypnosis, *t*(27) = 5.02, *p* < .001, *d* = 1.89 (Fig 2).

Although we counterbalanced the order of condition presentation, we evaluated its possible effect. The Order of condition presentation had no significant main effect (*F* = .69), but its interaction with Hypnotizability did *F*(1, 25) = 10.39, *p* = .004, η_*p*_^*2*^ = .29: Highs experienced themselves as being more deeply hypnotized if they underwent the control condition first (*M* = 8.61, *SD* = 2.09) rather than the hypnotic condition first (*M* = 5.79, *SD* = 1.46), *t*(13) = 3.03, *p* = .009, *d* = 1.57. Lows' experiences were not affected by the order of presentation, *t* < 1. There was no significant effect on Depth for the interaction between Condition and Order of presentation. *t* < 1. Hypnotic depth as a covariate did not make any difference to the results reported later so it was not analyzed further (see Table 1 for the descriptive statistics for the various tasks).

**Table 1:** Descriptive statistics for the tasks across levels of hypnotizability (Lows vs. Highs) and Condition (Control vs. Hypnosis).

Task	Variable	Lows		Highs	
		Control	Hypnosis	Control	Hypnosis
		*M* (*SD*)	*M* (*SD*)	*M* (*SD*)	*M* (*SD*)
Fixation	Blink rate (blinks/s)	0.17 (0.15)	0.16 (0.14)	0.17 (0.12)	0.13 (0.13)
	Pupil size (a. u.)	269 (79)	258 (69)	262 (64)	261 (65)
Smooth pursuit	Saccade count	25.32 (7.70)	25.76 (7.08)	20.40 (4.10)	22.86 (6.12)
OKN	Saccade count	16.64 (4.89)	15.56 (3.3)	17.66 (3.39)	17.34 (3.1)
	Saccade amplitude (^o^)	4.66 (2.39)	4.2 (1.09)	4.87 (1.21)	4.68 (0.97)
	Fixation duration (ms)	478 (218)	479 (111)	396 (160)	424 (72)
Saccade	Saccade amplitude (^o^)	11.62 (0.52)	11.39 (0.34)	11.19 (0.62)	10.74 (0.7)
Prosaccade	Correct responses (%)	88.56 (8.1)	85.46 (10.72)	91.73 (8.71)	90.87 (5.27)
	Saccade latency (ms)	249 (44)	252 (80)	223 (31)	239 (28)
Antisaccade	Correct responses (%)	74.04 (18.04)	77.37 (11.4)	74.11 (12.56)	77.66 (8.9)
	Saccade latency (ms)	250 (45)	273 (46)	266 (37)	280 (26)

a. u. = arbitrary units; ^o^ = degrees; ms = milliseconds

### The fixation task

Average BR and pupil size did not correlate significantly, *r*(22) = .33, *p* = .137. The main hypothesis was that BR would decrease during hypnosis only for Highs, and that they would exhibit smaller pupil size. For BR, there were no main effects for Condition, *F*(1, 22) = 1.10, *p* = .306, η_*p*_^*2*^ = .05, Hypnotizability, *F*(1, 22) = 0.13 *p* = .725, η_*p*_^*2*^ = .01, or their interaction, *F*(1, 22) = 0.24, *p* = .629, η_*p*_^*2*^ = .01. Likewise, there were no significant main effects on pupil size for Condition, *F*(1, 22) = 0.31, *p* = .586, η_*p*_^*2*^ = .01, Hypnotizability, *F*(1, 22) = 0.01, *p* = .936, η_p_^2^ = .00, or their interaction, *F*(1, 22) = 0.21, *p* = .650, η_*p*_^*2*^ = .01.

### The smooth pursuit task

Participants on average made 46.87 saccades (*SD* = 11.41) across the two conditions. For saccades, there were no main effects for Condition, *F*(1, 24) = 1.37, *p* = .254, η_*p*_^*2*^ = .05, Hypnotizability, *F*(1, 24) = 3.32, *p* = .08, η_*p*_^*2*^ = .12, or their interaction, *F*(1, 24) = 0.66, *p* = .42, η_*p*_^*2*^ = .03. After adding sex as a covariate, there was an interaction between sex and condition, *F*(1, 22) = 6.27, *p* = .020, revealing only a nonsignificant tendency for males to produce more saccades in the hypnosis than the control condition, *F*(1, 10) = 3.96, *p* = .075. With respect to the hypothesis that dissociation would lead to detachment from the task and catching-up saccades, there was no significant correlation between dissociation and total saccades, *r*(24) = -.35, *p* = .084.

### The optokinetic nystagmus task (OKN)

The hypothesis that during hypnosis Highs would produce fewer saccades and lower saccade amplitude than Lows was not supported. Participants executed on average 16.91 saccades per trial (*SD* = 3.23), which did not vary significantly by Condition, *F*(1, 24) = 1.01, *p* = .325, η_*p*_^*2*^ = .04, Hypnotizability, *F*(1, 24) = 1.2, *p* = .285, η_*p*_^*2*^ = .048, or their interaction, *F*(1, 24) = 0.3, *p* = .59, η_*p*_^*2*^ = .012.

We next evaluated saccade amplitudes (*M* = 4.63 degrees, *SD* = 1.33). There were no main effects for Condition, *F*(1, 24) = 2.1, *p* = .161, η_*p*_^*2*^ = .08, Hypnotizability, *F*(1, 24) = 0.42, *p* = .522, η_*p*_^*2*^ = .02, or their interaction, *F*(1, 24) = 0.36, *p* = .554, η_*p*_^*2*^ = .02.

We also evaluated fixation duration, which had a mean of 439 ms (*SD* = 118). Duration did not relate to Condition, *F*(1, 24) = 0.37, *p* = .55, η_*p*_^*2*^ = .02, Hypnotizability, *F*(1, 24) = 2.24, *p* = .148, η_*p*_^*2*^ = .09, or their interaction, *F*(1, 24) = 0.38, *p* = .546, η_*p*_^*2*^ = .02. A participant had extremely long fixations during the control session (1s) and if we had removed these data Condition would have been significant *F*(1, 23) = 10.28, *p* = .004, η_*p*_^*2*^ = .31 with longer fixations in hypnosis (*M* = 440, *SD* = 89) than control (*M* = 406, *SD* = 97), but this would not have changed the results for Hypnotizability or its interaction with Condition.

### The saccade task

**Fig 3:**
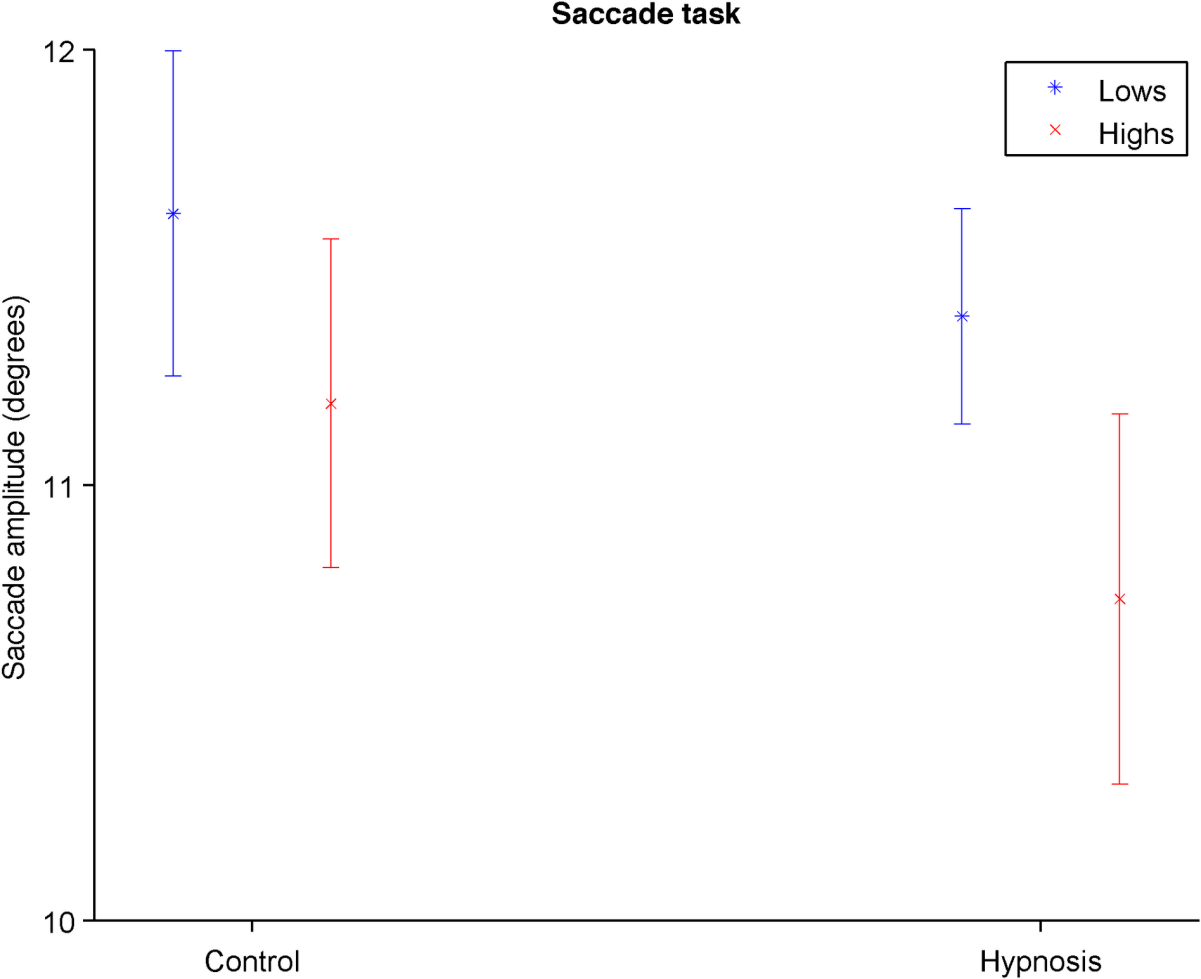
Mean saccade amplitude in the saccade task during the control and hypnosis conditions. Error Bars Indicate 95% Confidence Intervals.

Participants on average made saccades with an amplitude of 11.2 degrees (*SD* = 0.11). There was an overall effect for Condition, *F*(1, 21) = 7.42, *p* = .013, η_p_^2^ = .261, with the overall saccade amplitude decreasing under hypnosis (Fig 3). There was also a significant effect for Hypnotizability, *F*(1, 21) = 6.72, *p* = .017, η_p_^2^ = .24, with overall smaller saccade amplitudes for the Highs. There was no interaction between Condition and Hypnotizability, *F*(1, 21) = .72, *p* = .406, η_p_^2^ = .03.

### The antisaccade task

For *prosaccades*, participants on average had an average of 89% correct responses (*SD* = 8.43) with a saccade latency of 239.73 ms (*SD* = 48.31). The amount of correct responses did not vary by Condition, *F*(1, 27) = 0.96, *p* = .337, η_*p*_^*2*^ = .03, Hypnotizability, *F*(1, 27) = 3.34, *p* = .079, η_*p*_^*2*^ = .11, or their interaction, *F*(1, 27) = 0.31, *p* = .584, η_*p*_^*2*^ = .01. Saccade latency did not vary by Condition, *F*(1, 27) = 0.95, *p* = .34, *η*_*p*_^*2*^ = .0.3, Hypnotizability, *F*(1, 27) = 1.57, *p* = .221, *η*_*p*_^*2*^ = .06, or their interaction, *F*(1, 27) = 0.43, *p* = .517, *η*_*p*_^*2*^ = .02.

**Fig 4:**
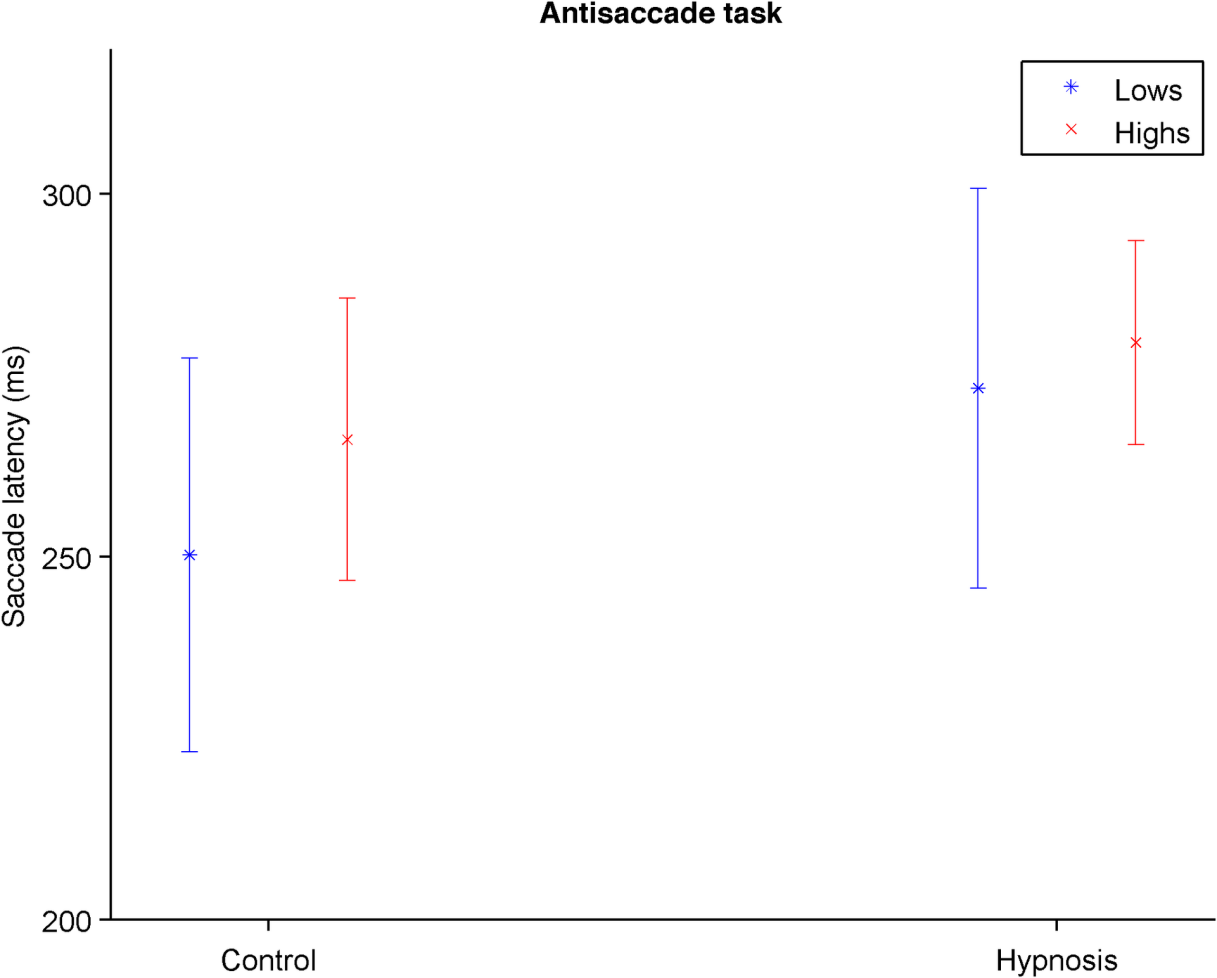
Mean saccade latency responding to antisaccades trials in the antisaccade task for control and hypnosis conditions across all participants. Error Bars Indicate 95% Confidence Intervals.

For *antisaccades*, participants on average responded correctly in 76% (*SD* = 13) of the trials with a saccade mean latency of 268 ms (*SD* = 39). The amount of correct responses in the antisaccade task did not vary significantly by Condition, *F*(1, 27) = 1.63, *p* = .213, η_*p*_^*2*^ = .06, Hypnotizability, *F*(1, 27) < 0.01, *p* = .964, η_*p*_^*2*^ < .01, or their interaction, *F*(1, 27) < 0.01, *p* = .969, η_*p*_^*2*^ < .01. The saccade latency, however, varied by Condition, *F*(1, 27) = 7.32, *p* = .012, η_*p*_^*2*^ = .21, with participants responding more slowly during hypnosis (Fig 4). However, there were no significant effects for Hypnotizability, *F*(1, 27) = 0.77, *p* = .387, η_*p*_^*2*^ = .03, or its interaction with Condition, *F*(1, 27) = 0.51, *p* = .481, η_*p*_^*2*^ = .02.

## Discussion

As expected, Highs experienced greater hypnotic depth during hypnosis (but not during control) than Lows, in agreement with various studies showing that a hypnotic procedure affects the conscious experience of Highs but not Lows [24]. Although this result is not novel it is useful for testing the other hypotheses as it shows that the hypnotic protocol had the expected effect. There was also an Order x Hypnotizability interaction effect, with Highs giving higher depth ratings when the control session preceded the hypnosis one. We had not hypothesized an order effect but this result is consistent with a study (see study 1 in [46]), in which participants were more responsive to hypnotic suggestions when they were presented after the non-hypnotic one but not vice versa.

With regard to the eye-tracking tasks, we did not replicate most of the results reported previously and only replicated others partially. The overall pattern of results did not support the assumption that specific eye behaviors are manifested only by Highs in hypnosis, not replicating the proposal that they can serve as an indicator of hypnosis.

Now, to specific results. First, for the *fixation* task, we found no significant differences for BR, in contrast with [1], and with group responses of lower BR among Highs [12, 16, 19]. Nor did we find in the *fixation* task a smaller pupil size among Highs, in contrast with [1]. For the *smooth pursuit*, and the *OKN* tasks, there were no significant differences in frequency of saccades, nor were there differences in *OKN* for saccade amplitude or fixation duration (in the latter, removing an outlier would have made Condition significant, but this was a post-hoc analysis that would need to be replicated). In contrast, Kallio et al. [1] reported that their virtuoso in the *OKN* test had a decrease in the number, size, and speed of ocular movements, and a decrease of eye fixation.

For the remaining tasks, there were a few significant main effects, but no significant interactions. For the *saccade* task there were smaller saccades for both groups during hypnosis, which would support the proposal of reduced eye mobility during hypnosis [12, 14], but because they were manifested by both groups they probably have more to do with other variables associated with undergoing a hypnotic condition (e.g., demand characteristics or relaxation in general) than with being hypnotized *per se* [3]. Thus, they do not constitute a replication of the short saccades shown in [1]. Something similar could be said of the antisaccades result that both Highs and Lows responded more slowly during hypnosis; the lack of a significant interaction in this task does not support the hypothesis of reduced prefrontal control of Highs during hypnosis [32].

There were results of marginal significance that might have become significant in a study with greater power. Although not significant, the size of the *r* = .35 correlation between dissociative tendencies and saccades in the smooth pursuit task is sizable enough that it would be worth revisiting in a study with a larger *N* that also includes clinically dissociative individuals.

In sum, overall we did not replicate some earlier findings or the proposal of Kallio et al. [1] that specific eye behaviors as measured by eye-tracking tasks can be used as indicators of hypnosis. Nonetheless our results must be placed in context. First, our study does not refute the unusual ability of Kallio et al.'s participant to control her ocular behavior, but it does not confirm their conclusion that this ability is an index of high hypnotizability. It is entirely plausible that their participant might differ from all other/most/some/a few Highs in other relevant characteristics than hypnosis. Second, both Kallio et al.'s and our study evaluated the ocular behavior of participants during intrusive tests, but they did not evaluate spontaneous ocular behavior after a hypnotic induction. Weitzenhoffer reported in various studies that there is such a thing as a "hypnotic stare,” and he may be correct for spontaneous behavior in the absence of intrusive tests, although our results suggest that these purported changes may have more to do with being in a hypnotic condition than with the performance of (only) Highs in hypnosis. It makes sense that a condition that asks participants to focus on their inner experiences might produce reduced ocular scanning of the environment. This hypothesis should be investigated further, including comparing an eye fixation with other types of hypnotic inductions.

Limitations of our study include a relatively small *N*, although the overall similarity of the Means in the results suggests that significant differences, if any, found in a higher-powered study are likely to be small. Our Highs group did not include only people scoring 12 in a hypnotizability scale as with Kallio et al.'s participant, but they are representative of the general population of Highs, and the Highs included respondents scoring 11 and 12, which can be passed only by those reporting such phenomena as hallucinations and/or amnesia. Although we did not test all of our sample with a more rigorous hypnotizability test (such as [47]), we did test three of them for another project and all of them remained Highs in that test as well. Furthermore, the results using the *Subjective Experiences Scale* confirmed that the Highs were not merely behaviorally compliant but were subjectively much more affected by the suggestions than the Lows, which the reports for hypnotic depth in our study corroborated. As Register and Kihlstrom concluded (p. 84), "Assessments of hypnotizability are enhanced when investigators consider subjective involvement as well as behavioral measures" [48].

Our results do not preclude the possibility that in a more spontaneous or different setting the experience of Highs of being hypnotized might relate to specific eye behaviors. Furthermore, any potential relation between eye behavior and hypnosis may be mediated/moderated by other variables such as dissociation [48–49]. Some authors [12, 14] who reported a relation between eye behaviors and hypnosis also mentioned considerable variability, so future studies might consider investigating how individuals in hypnosis who show marked eye behavior changes differ from their counterparts, rather than just using aggregate analysis stratified by hypnotizability, or assuming that any index of the latter will apply to all (or even most) of the members of that category. Our results indicate that, if any, the relation between hypnosis, hypnotizability, and eye behavior is more complex and nuanced than previously asserted and will require careful investigation.

## References

[1] Kallio S, Hyönä J, Revonsuo A, Sikka P, Nummenmaa L. The existence of a hypnotic state revealed by eye movements. PloS ONE. 2011;6: e26374 doi: 10.1371/journal.pone.0026374 22039474 10.1371/journal.pone.0026374 PMC3200339

[2] Kirsch I, Lynn SJ. Altered state of hypnosis: Changes in the theoretical landscape. Amer Psychologist. 1995;50:846–858.

[3] Kihlstrom J. The domain of hypnosis. revisited In Nash MR, Barnier AJ, editors. The Oxford handbook of hypnosis: Theory, research and practice. Oxford, UK: Oxford University Press, 2008, pp. 21–52.

[4] Charcot JM, Richer P. Les démoniaques dans l'art (The possessed in art). Amsterdam, The Netherlands: B. M. Israel; 1972, originally published 1887.

[5] Sargant W. Battle for the mind. New York, NY: Doubleday 1957.

[6] Sargant W. The mind possessed: A physiology of possession, mysticism and faith healing. Philadelphia, PA: J. P. Lippincot 1973.

[7] Ellenberger HF. The discovery of the unconscious. New York, NY: Basic Books 1970.

[8] Yeates LB. James Braid: Surgeon, gentleman scientist, and hypnotist Doctoral dissertation. University of New South Wales, Australia 2013

[9] Spiegel H, Spiegel D. Trance and treatment: Clinical uses of hypnosis. New York, NY: Basic Books 1978.

[10] Sheehan DV, Latta WD, Regina EG, Smith GM. Empirical assessment of Spiegel's Hypnotic Induction Profile and eye-roll hypothesis. Int J Clin Exp Hypn. 1979;27:103–110. doi: 10.1080/00207147908407550 541128 10.1080/00207147908407550

[11] Weitzenhoffer A. Eye-blink rate and hypnosis: Preliminary findings. Percept Mot Skills. 1969a;2:671–676. 10.2466/pms.1969.28.2.671 5803494

[12] Weitzenhoffer A. Ocular changes associated with passive hypnotic behavior. Am J Clin Hypn. 1971;14:102–121. doi: 10.1080/00029157.1971.10402158 5163593 10.1080/00029157.1971.10402158

[13] Weitzenhoffer A. Hypnosis and eye movements. I. Preliminary reports on a possible slow eye movement correlate of hypnosis. Am J Clin Hypn. 1969b;11 221–227. doi: 10.1080/00029157.1969.10402041 5767560 10.1080/00029157.1969.10402041

[14] Tebecis AK, Provins KA. Hypnosis and eye movements. Biol Psychol. 1975;3: 31–47. 169921 10.1016/0301-0511(75)90004-6

[15] Dunwoody RC, Edmonston WE. Hypnosis and slow eye movements. Am J Clin Hypn. 1974;16:270–274.

[16] Lindsay S, Kurtz RM, Stern JA. Hypnotic susceptibility and the endogenous eye-blink: A brief communication. Int J Clin Exp Hypn. 1993;41:92–96. doi: 10.1080/00207149308414540 8468106 10.1080/00207149308414540

[17] Lichtenberg P, Even-Or E, Bachner-Melma R, Levin R, Brin A, Heresco-Levy U. Hypnotisability and blink rate: A test of the dopamine hypothesis. Int J Clin Exp Hypn. 2008;56 243–254. doi: 10.1080/00207140802039474 18569136 10.1080/00207140802039474

[18] Eckstein MK, Guerra-Carrillo B, Miller Singley AT, Bunge SA. Beyond eye gaze: What else can eyetracking reveal about cognition and cognitive development? Dev Cogn Neurosci. 2016 doi: 10.1016/j.dcn.2016.11.001 27908561 10.1016/j.dcn.2016.11.001 PMC6987826

[19] Di Gruttola F, Orsini P, Carboncini MC, Rossi B, Santarcangelo EL. Revisiting the association between hypnotisability and blink rate. Exp Brain Res. 2014;23:3763–3769. 10.1007/s00221-014-4073-z 25138913

[20] Smilek D, Carriere JSA, Cheyne JA. Out of mind, out of sight: Eye blinking as indicator and embodiment of mind wandering. Psychol Sci. 2010;21:786–789. doi: 10.1177/0956797610368063 20554601 10.1177/0956797610368063

[21] Cardeña E, Marcusson-Clavertz D. The relation of hypnotizability and dissociation to everyday mentation: An experience sampling study. Psychol Conscious. 2016;3:61–79.

[22] Aston-Jones G, Cohen JD. An integrative theory of locus coeruleus- norepinephrine function: adaptive gain and optimal performance. Annu Rev Neuroscience. 2005;28:403–450. 10.1146/annurev.neuro.28.061604.135709 16022602

[23] Laeng B, Sirois S, Gredebäck G. Pupillometry a window to the preconscious? Perspect Psychol Sci. 2012;7(1):18–27. doi: 10.1177/1745691611427305 26168419 10.1177/1745691611427305

[24] Cardeña E, Jönsson P, Terhune DB, Marcusson-Clavertz D. The neurophenomenology of neutral hypnosis. Cortex. 2013;49: 375–385. doi: 10.1016/j.cortex.2012.04.001 22579225 10.1016/j.cortex.2012.04.001

[25] McGeown WJ, Mazzoni G, Venneri A, Kirsch I. Hypnotic induction decreases anterior default mode activity. Conscious Cogn. 2009;18:848–855. doi: 10.1016/j.concog.2009.09.001 19782614 10.1016/j.concog.2009.09.001

[26] Cardeña E. Altering consciousness: Setting up the stage In Cardeña E, Winkelman M, editors. Altering consciousness. Multidisciplinary perspectives. Volume I. History, culture, and the humanities. Santa Barbara, CA: Praeger; 2011 pp. 1–21.

[27] Kallio S, Koivisto M. Seeing blue as red: A hypnotic suggestion can alter visual awareness of colors. Int J Clin Exp Hypn. 2016;64:261–284. doi: 10.1080/00207144.2016.1171088 27267673 10.1080/00207144.2016.1171088

[28] Terhune DB, Cardeña E. Nuances and uncertainties in hypnotic inductions: Towards a theoretically informed praxis. Am J Clin Hypn. 2016; 59:155–174. doi: 10.1080/00029157.2016.1201454 27586045 10.1080/00029157.2016.1201454

[29] Levy DL, Mendell NR, Holzman PS. The antisaccade task and neuropsychological tests of prefrontal cortical integrity in schizophrenia. Empirical findings and interprettive considerations. World Psychiatry. 2004;3:32–40. 16633452 PMC1414662

[30] Deuter CE, Schilling TM, Kuehl LK, Blumenthal TD, Schachinger H. Startle effects on saccadic responses to emotional target stimuli. Psychophysiology. 2013;50:1056–1063. doi: 10.1111/psyp.12083 23841560 10.1111/psyp.12083

[31] Crawford HJ, Horton JE, Harrington GS, Downs III JH. Attention and disattention (hypnotic analgesia) to noxious somatosensoty TENS stimuli: fMRI differences in low and highly hypnotizable individuals. Neuroimage. 2000;11 doi: 10.1016/S1053-8119(00)90978-9

[32] Woody EZ, Bowers SA frontal assault on dissociated control In Lynn SJ, & Rhue JW, editors. Dissociation: Clinical and theoretical perspectives. New York, NY: Guilford 1994 pp. 52–79.

[33] Hutton SB. Cognitive control of saccadic eye movements. Brain Cogn. 2008;68:327–401. doi: 10.1016/j.bandc.2008.08.021 19028265 10.1016/j.bandc.2008.08.021

[34] Liversedge SP, Findlay JM. Saccadic eye movements and cognition. Trends Cogn Sci. 2000;4:6–14. 10637617 10.1016/s1364-6613(99)01418-7

[35] Irwin HJ, Green MJ, Marsh PJ. Dysfunction in smooth pursuit eye movements and history of childhood trauma. Percept Mot Skills. 1999;8:1230–1236. 10.2466/pms.1999.89.3f.1230 10710773

[36] O’Driscoll GA, Callahan BL. Smooth pursuit in schizophrenia: A meta- analytic review of research since 1993. Brain Cogn. 2008;68:359–370. doi: 10.1016/j.bandc.2008.08.023 18845372 10.1016/j.bandc.2008.08.023

[37] Brainard DH. The Psychophysics Toolbox. Spat Vis. 1997;10:433–436. 9176952

[38] Cornelissen FW, Peters EM, Palmer J. The Eyelink Toolbox: Eye tracking with MATLAB and the Psychophysics Toolbox. Behav Res Methods, Instruments, Comput. 2002;34:613–617. 10.3758/bf03195489 12564564

[39] Shor RE, Orne EC. Harvard Group Scale of Hypnotic Susceptibility: Form A. Palo Alto, CA: Consulting Psychologists Press 1962.

[40] Council J. Measures of hypnotic responding In Kirsch I, Capafons A, Cardeña-Buelna E. Amigó S, editors. Clinical hypnosis and self-regulation: Cognitive-behavioral perspectives. Washington, DC: American Psychological Association, 1999, pp. 119–140.

[41] Evans FJ, Schmeidler D. Relationship between the Harvard Group Scale of Hypnotic Susceptibility and the Stanford Susceptibillity Scale: Form C. Int J Clin Exp Hypnosis. 1966;(4):333–343. 10.1080/00207146608412976 5977013

[42] Kirsch I, Council JR, Wickless C. Subjective scoring for the Harvard Group Scale of Hypnotic Susceptibility, Form A. Int J Clin Exp Hypn. 1990;38:112–124. doi: 10.1080/00207149008414506 2347668 10.1080/00207149008414506

[43] Cardeña E. & Terhune D. B. (2014). Hypnotizability, personality traits, and the propensity to experience alterations of consciousness. *Psychology of Consciousness*: *Theory*, *Research*, *and Practice*, 1, 292–307.

[44] Carlson EB, Waelde LC, Palmieri PA, Macia KS, Smith SR, McDade-Montez E. Development and validation of the Dissociative Symptoms Scale. Assessment. doi: 10.1177/1073191116645904 27178761 10.1177/1073191116645904

[45] Laurence JR, Nadon R. Reports of hypnotic depth: Are they more than mere words? Int J Clin Exp Hypnosis. 1986;(34):215–233. 10.1080/00207148608406987 3744614

[46] Braffman W, Kirsch I. Imaginative suggestibility and hypnotizability: An empirical analysis. J Pers Soc Psych. 1999;77:577–587. 10.1037//0022-3514.77.3.578 10510510

[47] Weitzenhoffer AM, Hilgard ER. Stanford Hypnotic Susceptibility Scale, Form C. Palo Alto, CA: Consulting Psychologists Press 1962.

[48] Register PA, Kihlstrom JF. Finding the hypnotic virtuoso. Int J Clin Exp Hypn. 1986;34:84–97. doi: 10.1080/00207148608406974 3699937 10.1080/00207148608406974

[49] Terhune DB, Cardeña E., Lindgren M. Dissociative tendencies and individual differences in high hypnotic suggestibility. Cogn Neuropsychiatry. 2011;16:113–135. doi: 10.1080/13546805.2010.503048 20721761 10.1080/13546805.2010.503048

